# Percutaneous Alginate Hydrogel Endomyocardial Injection with a Novel Dedicated Catheter Delivery System: An Animal Feasibility Study

**DOI:** 10.1007/s12265-024-10497-8

**Published:** 2024-02-20

**Authors:** Bo Wang, Chao Gao, Scott Lim, Rutao Wang, Cun-jun Zhu, Yoshinobu Onuma, Yunbing Wang, Runlin Gao, Patrick W. J. C. Serruys, Randall J. Lee, Ling Tao

**Affiliations:** 1 Department of Cardiology, Xijing Hospital, Fourth Military Medical University, Xi’an, China; 2 Corrib Research Centre for Advanced Imaging and Core Laboratory, University of Galway, Galway, Ireland; 3 Department of Medicine, Division of Cardiovascular Medicine, University of Virginia, Charlottesville, VA USA; 4 National Engineering Research Center for Biomaterials, Sichuan University, Sichuan, China; 5 Fuwai Hospital, Chinese Academy of Medical Sciences & Peking Union Medical College, Beijing, China; 6 Department of Medicine, University of California-San Francisco, San Francisco, CA USA

**Keywords:** Alginate hydrogel, Biomaterial, Percutaneous treatment

## Abstract

The objective of this preclinical study was to evaluate the feasibility and safety of transcatheter endocardial alginate hydrogel injection (TEAi) in a large animal model, utilizing the high-stiffness XDROP® alginate hydrogel in combination with the dedicated EndoWings® catheter-based system. All swine (*n* = 9) successfully underwent TEAi without complications. Acute results from a subset of animals (*n* = 5) demonstrated the ability of the catheter to access a wide range of endomyocardial areas and achieve consecutive circumferential hydrogel distribution patterns within the mid-left ventricular wall. Histological examinations at 6 months (*n* = 4) demonstrated that the XDROP® remained localized within the cardiac tissue. In addition, serial echocardiographic imaging showed that XDROP® had no adverse impacts on LV systolic and diastolic functions. In conclusion, this innovative combination technology has the potential to overcome the translational barriers related to alginate hydrogel delivery to the myocardium.

**Graphical abstract:**

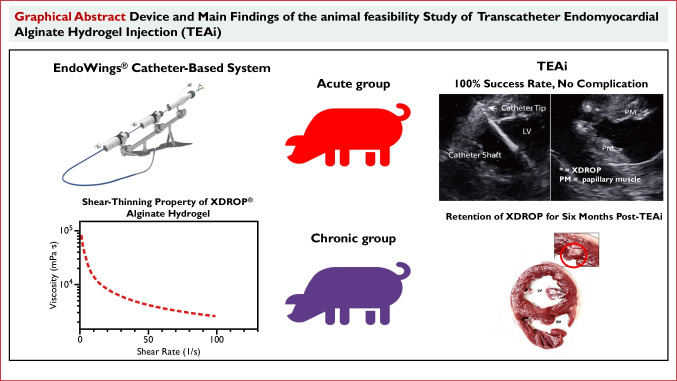

**Supplementary Information:**

The online version contains supplementary material available at 10.1007/s12265-024-10497-8.

## Introduction

Heart failure (HF) is a leading cardiovascular disease with a 5-year mortality rate of approximately 50% [1]. The current definitive treatment for end-stage HF is heart transplantation, while left ventricular assist devices (LVADs) serve as a bridge to transplantation [2]. However, these approaches are severely limited by organ scarcity, prolonged dependence on external devices, and the risks of invasive surgery.

In recent years, intramyocardial injection of acellular alginate hydrogels (AHs) has emerged as a promising alternative for treating HF [3]. This therapy aims to reinforce the compromised left ventricular wall and reduce wall stress, potentially providing valuable clinical benefits [4]. The pursuit of clinical translation for this treatment has led to a series of investigations involving the optimization of alginate hydrogel properties, injection patterns, and delivery routes [5–8]. Several studies have used computational finite element models to demonstrate the importance of stiffness augmentation of implants and consecutive circumferential mid-left ventricular (LV) wall injection patterns in reducing wall stress [9–13]. Meanwhile, there is a growing consensus in favor of minimally percutaneous invasive procedures due to their lower risk profile than open-chest procedures or intracoronary infusion of the hydrogel [14]. Thus, transcatheter endomyocardial alginate hydrogel injection (TEAi) is emerging as a promising solution; however, its practical application is challenged by the inherent high viscosity of alginate hydrogels, leading to catheter lumen occlusion, and the requirement for facile catheter manipulability [15].

To address this gap, we have developed an injectable high-stiffness alginate hydrogel with concomitant shear-thinning properties engineered to diminish viscosity during catheter-based delivery. In addition, we have introduced a dedicated catheter-based injection system designed to implement consecutive circumferential mid-ventricular wall injection (the EndoWings® system), mimicking the AH distribution patterns in surgical procedures. This study aims to evaluate the feasibility and safety of this novel technology combination in a preclinical swine model. This approach offers the promise of catheter-based therapy that can be used in HF patients to attenuate the remodeling process and prevent mortality.

## Methods

### In Vitro XDROP® Evaluation

To assess the properties of XDROP®, in vitro studies were conducted to evaluate several parameters, including the swelling coefficient, weight-averaged molecular weight, mechanical properties, injectability, and hydrogel morphology. Alginate samples were performed thrice to obtain the average value for each in vitro test. For swelling coefficient, the pre-weighed dry hydrogels were immersed in distilled water at 37 ℃, and their weight change was monitored at different intervals until the hydrogels showed complete dissolution. The swelling coefficient was defined as follows:

$$\mathrm{Swelling\;coefficient\;}\left(\mathrm{\%}\right)=[(\mathrm{Final\;weight}-\mathrm{Initial\;weight})/\mathrm{ Initial\;weight}]*100$$

The weight-averaged molecular weight distribution of alginate hydrogel was measured on an aqueous gel permeation chromatography, GPC (H20, Waters) system.

For mechanical properties, rheological experiments were conducted in an Anton Paar MCR 302 rheometer using a cone-and-plate geometry with a diameter of 25 mm. The sample was applied to the plate. The test parameters of the mechanical property were *γ* = 0.5%, *f* = 1 Hz, and *T* = 20 ℃.

### EndoWings® Design Features

**Fig. 1:**
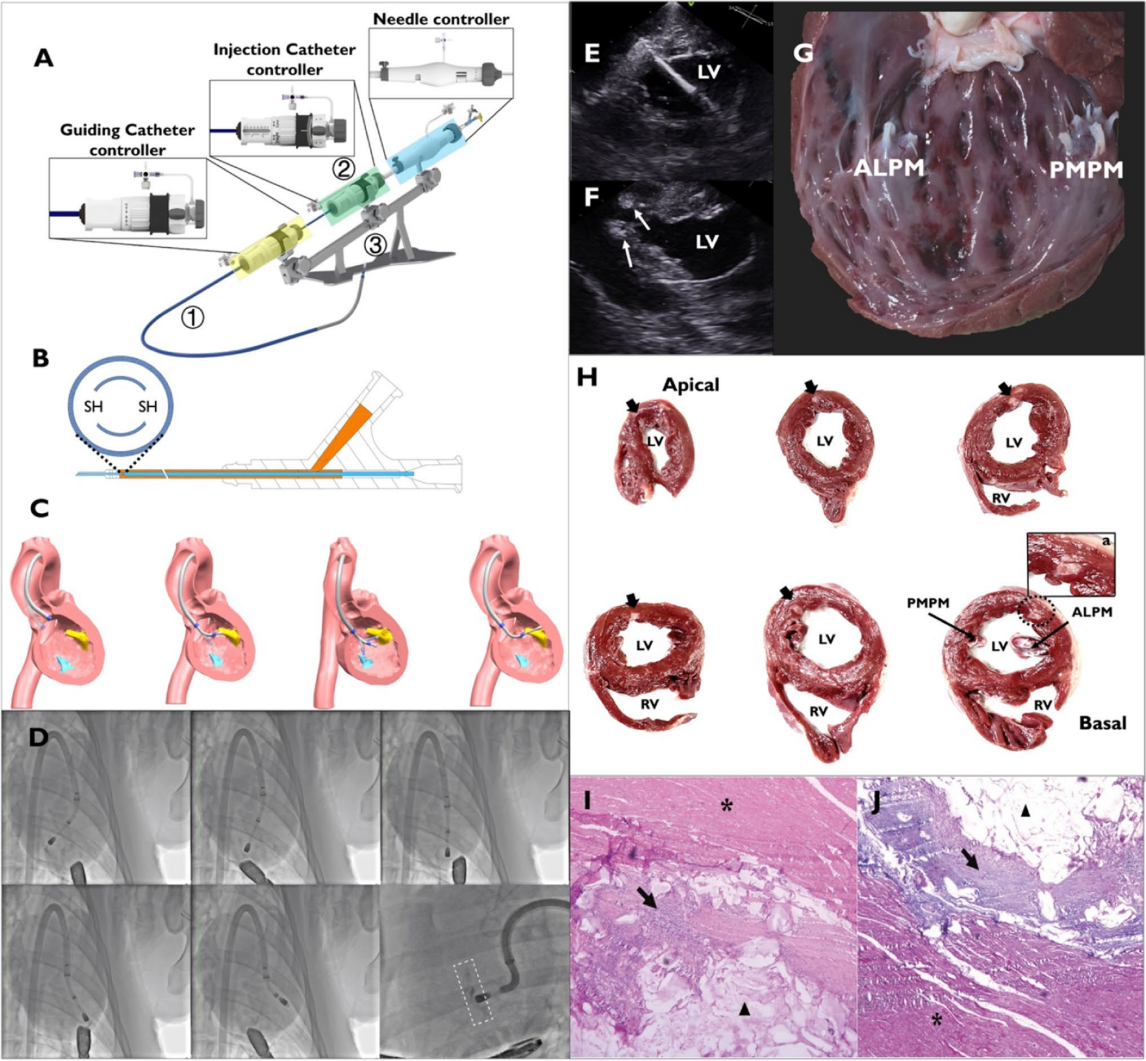
Illustrates the design and performance of the EndoWings® in vivo. **A** In this panel, ① is the 18-F guiding catheter (GC) and its controller (enclosed in a yellow frame). ② is the injection catheter (IC), specifically designed with a dedicated controller (enclosed in a green frame) to deliver therapeutic agents to the target anatomical site. The needle’s advancement and retraction are facilitated through a rotating wheel (enclosed in a blue frame), and the depth can be accurately specified via a distance monitoring window. The proximal end of the IC is connected to a syringe containing the treatment material and contrast medium. The stabilizer ③ enables the precise manipulation of the catheter and maintains stable endomyocardial contact during the procedure. Its primary function is to reduce the need for an interventional cardiologist to manually hold the catheter, allowing more accurate and consistent catheter movements. **B** This panel shows the IC, which features a double-lumen microcatheter with an outer diameter of 0.97 mm and an inner diameter of 0.81 mm and is equipped with a 25-gauge retractable needle. The design of this dual lumen microcatheter features a concentric circular structure with a side hole (SH) at the distal end of the inner lumen, facilitating the injection of contrast agent. **C** This section demonstrates the multiple orientation capacity of EndoWings®. The distal part of the GC can be oriented anteriorly or posteriorly. The distal portion of the IC is manipulated using a dedicated controller that enables deflection (straightening and bending) and steering (anterior to posterior) functionalities. Once the distal end of the catheter is directed toward the target endocardial site, the distal portion of the IC can be advanced to the LV wall. **D** Under fluoroscopy, the manipulated catheter tip can reach from the anterior to the posterior wall of the left ventricle and from the apical to the midventricular region, achieving consecutive circumferential LV-mid wall injections (enclosed in dots line frame). **E** The short axis of the echocardiogram clearly shows the relationship between the catheter and the wall of the left ventricle. **F** the deposition of the XDROP® (white arrows) in the myocardium. **G** Macroscopic evaluation shows the distribution of the injection site within the endocardium. **H** Six months post-injection, a gross anatomical examination reveals the retention of XDROP® (black arrows) in the myocardium. **I** Hematoxylin and eosin staining of the heart tissue showed slight multinucleate giant cell infiltration (black arrow) in the alginate hydrogel area (triangle) and no abnormalities in the surrounding myocardium (asterisk) 6 months after the procedure. **J** Masson trichrome staining showing encapsulation of the hydrogel (triangle) by fibrosis (black arrow). ALPM, anterolateral papillary muscle; LV, left ventricle; PMPM, posterior media papillary muscle; RV, right ventricle. SH, side hole

The EndoWings® catheter system was built with the assistance of Deke MedTech. It comprised three components: a guiding catheter (GC), an injection catheter (IC), and a stabilizer. The system layout and a schematic illustration of catheter manipulation are shown in Fig. 1A–C and Supplemental video.

### Injectability and Compatibility

The force used to inject the XDROP® via the EndoWings® was measured by the universal testing machine (UTM4103, SUNS, China). The test rate was 30 mm/min, equivalent to 1 mL/100 s. The maximum force per injection was recorded.

In vitro simulation tests were designed to measure the changes in mechanical properties of the XDROP® before and after delivery through the EndoWings®, including the change in swelling coefficient and the ratio of loss modulus to storage modulus (G''/G'). Scanning electron microscopy (SEM) examined the microstructure of the XDROP® before and after delivery through the EndoWings®.

### XDROP® Aggregation Experiment

Swine EDTA plasma was activated by adding 25 μL of physiological saline containing CaCl_2_ (0.5 mol/L) to each milliliter of plasma [16]. Two PVC tubes (30 cm) with an inner diameter of 3 mm were used, and the circulation speed of the peristaltic pump was set at 64 mL/min to simulate the flow of human blood (approximately 15 cm/s). Three milliliters of XDROP® alginate hydrogel was added to 100 mL of simulated blood fluid, and the hydrogel was uniformly dispersed under low-speed magnetic stirring. A peristaltic pump circulated the entire solution containing the hydrogel for 1 h, 12 h, and 24 h. PVC tubes were wrapped with a polyetheretherketone (PEEK) filter (pore size 115 × 145 μm, similar to TriGUARD 3 cerebral protection device) to capture the potential embolic particles. The number of hydrogel clot particles in the filter was recorded and counted using a microscope (VMS322, Henglei Guangdian Technology Co., Ltd, Hangzhou, China).

### Animal Experiment Design

The Animal Care and Use Committee of Xijing Hospital approved the animal experiments and protocols. Nine swine subjects (weight 78–90 kg) were divided into two groups. One group (*n* = 5) was sacrificed immediately after injection to evaluate the feasibility and safety of TEAi; the other group (*n* = 4) was sacrificed at 6 months to investigate the retention of XDROP® in the myocardium and alginate embolization to other organs.

The feasibility was defined as the ability of the EndoWings® to inject XDROP® into a specific LV endocardial area (from the apex to the mid-LV wall and from the anterior to the posterior wall) and achieve a consecutive circumferential mid-LV wall injection pattern. Safety was evaluated based on animal mortality and adverse events. In chronic animals, neurological events were recorded, and electrocardiographic monitoring was performed before, during, and after the procedure to assess conduction abnormalities and arrhythmias.

### In Vivo Transcatheter Endomyocardial Alginate-Hydrogel Injection

The swine subject was sedated and placed on mechanical ventilation. Electrocardiograms were monitored throughout the procedure to detect arrhythmias. The left thoracotomy was performed, and the pericardial sac was removed to place the echo probe near the heart. An abdominal incision was made to expose the descending aorta. A needle was used to puncture the aorta and insert a 6-F sheath. A 0.035-inch guidewire (GW) was delivered into the LV, and the GC was advanced over the GW into the LV. The IC was inserted into the central lumen of the GC. Catheter manipulation was guided by echocardiography. The interventional cardiologist controlled the catheter and confirmed the tip position using echocardiography. The tip reached the LV wall and was detected by echocardiography in the presence of a transient premature ventricular beat. A retractable 25-gauge needle was extended approximately 5 mm into the myocardium and used to administer each injection lasting 30 s. The successful intramyocardial injection was marked by the appearance of a bright signal perpendicular to the needle tip relative to the LV wall. After the procedure completion, the catheter was removed, and the incisions in the chest and abdomen were closed.

### Echocardiography

In the acute group, which was euthanized post-operatively, transthoracic echocardiography was performed within 24 h before the procedure (baseline echocardiogram) and immediately after the operation. For the chronic group under follow-up, echocardiographic evaluations were conducted within 24 h before the procedure (baseline echocardiogram) and at 6 months post-injection. Echocardiographic assessments were performed using a commercial echocardiography system (ACUSON Cypress™ System), including ejection fraction (EF), end-diastolic diameter (EDD), end-systolic diameter (ESD), and the ratio of early to late diastolic transmitral flow velocity (E/A ratio).

### Gross and Histological Examinations

Animals in the acute-phase group were euthanized immediately after the injection. The hearts were extracted and dissected for direct visual confirmation of the injection sites. To evaluate chronic outcomes, the animals were sacrificed at 6 months. The LV was divided into slices along the short axis to assess alginate hydrogel retention. Histological analysis was performed on 0.5-mm-thick heart tissue slices stained with hematoxylin–eosin (H&E) and Masson’s trichrome to assess inflammation and fibrosis using light microscopy. Additionally, other organs were sectioned into 0.5-mm pieces for safety evaluation and examined by H&E staining.

### Statistical Analysis

Normally distributed data are presented as mean ± standard deviation and others as median and interquartile range. Normality was tested with the Kolmogorov–Smirnov test. A two-tailed paired *t*-test or independent sample *t*-test was used for normally distributed data to assess differences between groups or time points. The Mann–Whitney *U* test or Wilcoxon signed-rank test was used for data not following a normal distribution. Categorical variables were described as *n* (%). *p* < 0.05 was considered to be statistically significant. Statistical analysis was performed with IBM SPSS Statistics for Windows, version 28.0 (IBM Corp., Armonk, NY, USA).

## Results

### Properties of the XDROP® Hydrogel and Injectability

**Fig. 2:**
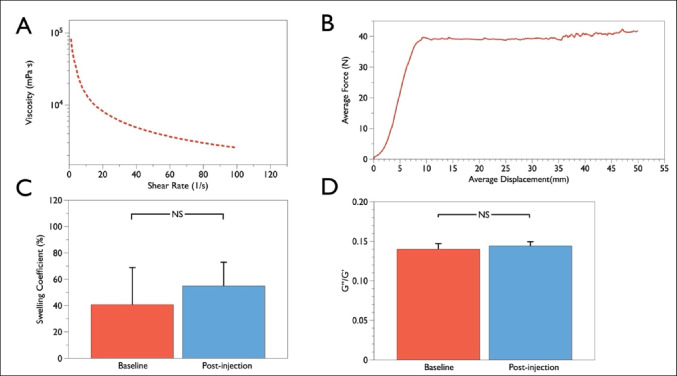
Assessments of XDROP®’s mechanical properties and injectability. **A** The relation between shear rate and viscosity of the hydrogel (*n* = 3), the y-axis is displayed as a logarithmic. **B** The force of the requirements for delivery of the XDROP® via EndoWings® (*n* = 3). **C** The change swelling coefficient before and after the injection (*n* = 3). **D** The change of G’’/G’ ratio before and after the injection (*n* = 3). G", storage modulus; G', loss modulus; NS represents the non-significant statistical difference

The weight average molecular weight of XDROP® was 12,389.67 ± 104.60 kDa. And the storage modulus (G') was in the range of 2–10 kPa. Figure 2A shows that the viscosity of the hydrogel decreased with increasing shear rate. This shear thinning behavior can be attributed to the ionic cross-linking within the hydrogel, which enhances its injectability. The injection force required to deliver the alginate hydrogel through the EndoWings® was also evaluated. The force increased to approximately 40 N at 10-mm injection depth and then plateaued (Fig. 2B).

The mechanical properties of the alginate hydrogel were evaluated before and after delivery through the EndoWings®. The results showed that there was no statistical difference in the swelling coefficient (40.71 ± 28.20 versus 54.82 ± 18.04, *p* = 0.374) and G''/G' (0.14 ± 0.01 versus 0.15 ± 0.01, *p* = 0.347) before and after delivery through the EndoWings® (Fig. 2C, D). SEM results showed no obvious change in the microstructure of XDROP® (Supplemental Fig. 1).

The aggregation experiment showed no solid particle was captured by the filter, indicating that the XDROP® hydrogel dispersed in simulated blood fluid would not aggregate into clots (Supplemental Fig. 2).

### In Vivo Evaluation of XDROP® Feasibility

TEAi were successfully performed in all swine with no procedural or device-related complications (*n* = 9). Under fluoroscopic and TEE guidance, we evaluated catheter maneuverability in the LV cavity. The results showed that the catheter had the required maneuverability to reach a wide area of the LV free wall from the apex to the mid-LV wall and from the anterior to the posterior LV wall and achieve consecutive circumferential mid-LV wall injections (Fig. 1D). TEE provided a clear visualization of the catheter position, its distal tip in relation to the ventricular wall, and the hydrogel distribution (Fig. 1E, F). Macroscopic evaluation post-injection shows injection sites located on the endocardial surface (Fig. 1G). The mean procedure time calculated from anesthesia was 114.8 ± 15.3 min. The mean number of injection sites was 10.0 ± 1.8, and the mean total injection volume was 3.0 ± 0.45 mL (Table 1).

**Table 1:** Animal demographics and procedural characteristics

Sex	Group	Body weight (kg)	Procedural time (min)	Total Injection volume (ml)	Total injection sites (*n*)
Female	Acute	85	135	3.3	11
Female	Acute	90	135	3	10
Male	Acute	89	95	2.7	9
Male	Acute	86	125	3.3	11
Female	Acute	88	110	3.6	12
Male	Chronic	85	98	3.3	11
Female	Chronic	82	115	2.7	11
Female	Chronic	84	100	3.0	9
Female	Chronic	78	120	2.1	6

### Safety Outcomes

No procedural or device-related complications, including death, cardiac injury, neurological abnormalities, or sustained ventricular arrhythmias, were observed from the time of injection to sacrifice.

### Gross and Histological Assessments

To assess the early inflammatory response post-injection, we performed histological evaluations of porcine myocardium 3 days after the injection of XDROP® (*n* = 3). The results showed a moderate mononuclear cell infiltration around the hydrogel, consisting mainly of lymphocytes and some macrophages, with no significant changes in the surrounding myocardial tissue. In addition, there was no apparent fibrous encapsulation around the hydrogel at 3 days post-implantation compared to 6 months post-implantation (Supplementary Fig. S3). Figure 1H illustrates the chronic outcome, in which swine was sacrificed at 6 months post-operatively to show the retention of XDROP® within the myocardium. H&E staining revealed a mild infiltration of multinucleated giant cells in the alginate-hydrogel region, with no abnormality in the surrounding myocardium (Fig. 1I). Masson’s trichrome staining demonstrated that the alginate-hydrogel was encircled by a fibrous layer (Fig. 1J). Gross morphometric examination revealed no pathological abnormalities in the kidneys, spleen, brain, liver, and lungs (Supplementary Fig. S4 and S5).

### Echocardiography Results

**Fig. 3:**
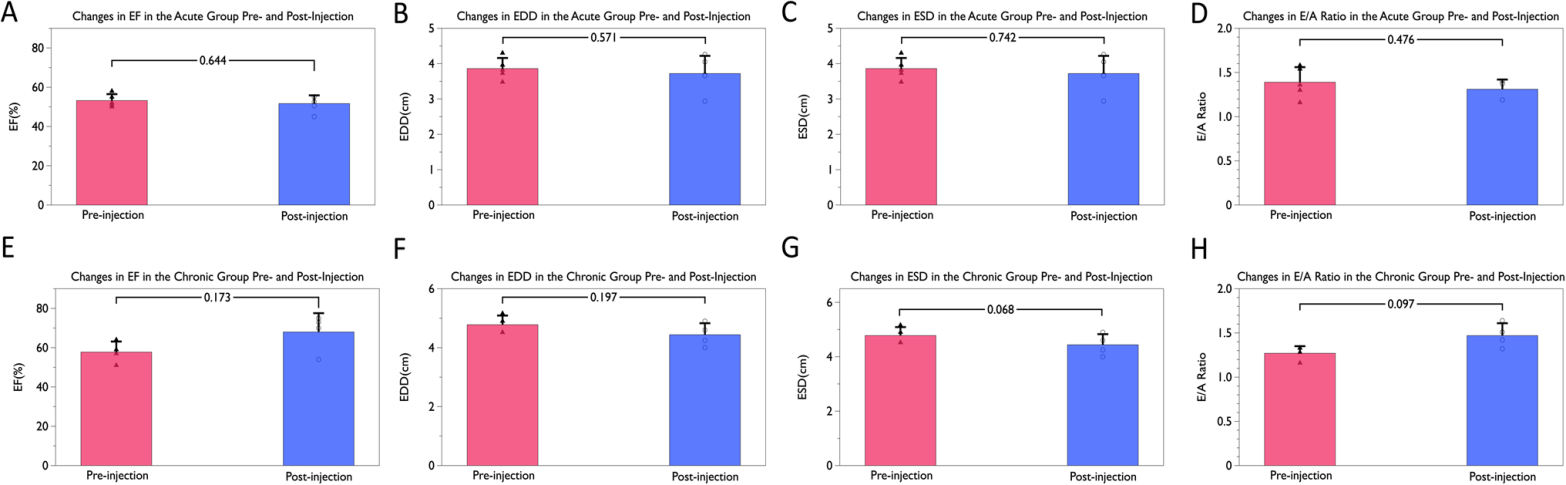
Changes in cardiac function as assessed by echocardiography before and after injection. **A** Changes in EF in the acute group. **B** Changes in EDD in the acute group. **C** Changes in ESD in the acute group. **D** Changes in E/A ratio in the acute group. **E** Changes in EF in the chronic group. **F** Changes in EDD in the chronic group. **G** Changes in ESD in the chronic group. **H** Changes in E/A ratio in the chronic group; E/A ratio = ratio of early to late diastolic transmitral flow velocity, EDD = end-diastolic diameter, EF = ejection fraction, ESD = end-systolic diameter

The echocardiographic findings indicated that there were no adverse effects of TEAi on cardiac systolic and diastolic function in both the acute and chronic groups. The results of the acute group were as follows: EF (53.2 ± 3.27% versus 51.7 ± 4.2%, *p* = 0.644), EDD (3.8 ± 0.3 cm versus 3.7 ± 0.5 cm; *p* = 0.571), ESD (2.8 ± 0.2 cm versus 2.6 ± 0.4 cm, *p* = 0.742), and E/A ratio (1.4 ± 0.2 versus 1.3 ± 0.1, *p* = 0.476). Similarly, the results of the chronic group were as follows: EF (57.8 ± 5.2% versus 68.0 ± 9.6%, *p* = 0.173), EDD (4.8 ± 0.3 cm versus 4.4 ± 0.4 cm; *p* = 0.197), ESD (3.3 ± 0.2 cm versus 2.5 ± 0.4 cm, *p* = 0.068), and E/A ratio (1.3 ± 0.1 versus 1.5 ± 0.1, *p* = 0.097). Figure 3 shows changes in cardiac function as assessed by echocardiography before and after injection.

## Discussion

In this study, we demonstrated the feasibility and safety of TEAi using the novel technological combination of the shear-thinning alginate hydrogel XDROP® with a dedicated EndoWings® system. Injectability and compatibility tests showed that the force required to inject XDROP® through the EndoWings® was achievable [17–19]. Mechanical properties of XDROP® after catheter delivery did not change significantly from baseline characteristics. Acute animal results showed that the catheter was able to reach a wide endomyocardial area and achieve consecutive circumferential mid-LV wall injection pattern, demonstrating the good maneuverability of the EndoWings®. No observations of adverse events support the safety of the TEAi. Gross examination confirmed the retention of XDROP® within the myocardium at 6 months post-procedure. These results suggest that the combination of XDROP® and EndoWings® can achieve results comparable to surgical open-chest injection methods [8].

The therapeutic mechanism of intramyocardial injection of AH aims to provide physical support to the cardiac tissue and increase the thickness of the LV wall, thereby reducing the wall stress and preventing adverse remodeling. To date, several studies have explored various injectable AH products for restoring cardiac function. BL-1040 is a form of aqueous calcium cross-linked alginate hydrogel (AH) that has been evaluated extensively in preclinical and clinical studies [5, 20]. This hydrogel has a viscosity ranging from 10 to 50 cP, making it a low-viscosity solution that is easily injectable through a catheter. The BL-1040 is distributed by the microvasculature and becomes deposited in the infarcted myocardium [7, 21]. Upon exposure to the increased calcium ion concentration in the infarcted tissue, the alginate biomaterial undergoes a phase transition to form a hydrogel in situ. While preclinical studies have shown promising results, subsequent phase II clinical trials have reported mixed outcomes regarding its efficacy in improving left ventricular enlargement and reducing cardiac events. The potential reason is that calcium ion concentration plays a key role in the gelation process of BL-1040. If the calcium ion concentration within the myocardium is uneven, this could lead to variations in the physical and chemical properties of the alginate hydrogel, differing from in vitro test results or expectations. Such heterogeneity could affect the mechanical stability of the hydrogel, degradation rate, and even its interaction with surrounding tissues, thereby impacting its effectiveness in improving left ventricular enlargement and reducing cardiac events.

Another injectable alginate hydrogel, Algisyl-LVR™, utilizes a two-component system that is mixed immediately prior to application. This hydrogel achieves rapid gelation within 3–4 min upon intramyocardial injection, exhibiting a mechanical strength of 3–5 kPa. Preclinical studies have indicated that circumferential mid-LV wall direct intramyocardial injections of Algisyl-LVR™ can reduce left ventricular (LV) volume and enhance ejection fraction (EF) [22]. The AUGMENT-HF trial further demonstrated improvements in exercise tolerance, symptoms, and overall clinical status in patients treated with this hydrogel [23]. However, there is a lack of data and research concerning the catheter-based delivery of Algisyl-LVR™. Given its rheological properties, the high viscosity of this hydrogel could pose challenges for catheter-based applications [3].

Compared to other biomaterials, the major advantages of the XDROP® lie in its pre-formed alginate hydrogel and shear-thinning properties. The pre-formed nature of the hydrogel ensures a well-defined structural quality and stability, allowing for rigorous quality control prior to application. This ensures the hydrogel meets the desired mechanical specifications and exhibits a high local retention rate within the myocardium. On the other hand, the shear-thinning property of the material enhances its injectability and adaptability. The viscosity of the hydrogel decreases under shear stress, making it easier to inject through needles and catheters. Once injected, the shear-thinning hydrogel quickly recovers its original viscosity, allowing for better integration with the surrounding tissue.

Various catheter-based transendocardial injection systems have been developed, primarily for delivering cells or therapeutic solutions [24–27]. Recently, VentriGel, an extracellular matrix (ECM) hydrogel derived from decellularized swine myocardium, has been validated for delivery using the MyoStar catheter [28]. However, to facilitate transendocardial delivery, VentriGel was engineered to have lower viscosity and moduli (10 Pa). These mechanical properties are insufficient to provide adequate tissue support, resulting in a different therapeutic mechanism than hydrogels with higher stiffness. Dolan et al. recently introduced an innovative advancement in catheter technology known as the advanced material catheter (AMCath) [17]. This specialized catheter is meticulously designed to efficiently administer rapidly gelling, covalently cross-linked hyaluronic acid hydrogels. A notable feature of this design is incorporating a reservoir within the catheter, where the hydrogel precursor solution undergoes thorough mixing immediately before injection through the needle. This innovative design significantly reduces the distance and time required to transport the hydrogel to the myocardium post-gelation, alleviating many challenges associated with the delivery process. However, it is important to acknowledge certain limitations of the AMCath system. Ensuring precise control over the hydrogel mixing process within the AMCath can be challenging, impacting the uniformity of hydrogel properties before and after injection. The mechanical properties of the hydrogels showed a reduction of up to 20.62% after passing through the AMCath.

Based on previous studies, in order to provide physical constraint function for enlarged LV, the mid-ventricular free wall serves as the target area for alginate hydrogel (AH) injection [13]. To mimic these methods, we identified the endocardial area between the anterior and posterior papillary muscles in the left ventricular free wall as the target area. To achieve this goal, the EndoWings® was designed with the following features: First, the system is equipped with independent, dedicated controllers for both the guide and injection catheters, allowing multidirectional adjustability. This design provides unprecedented flexibility for catheter movement within the complex anatomical structure of the heart, ensuring precise delivery of the therapeutic agent to the targeted anatomical site. Second, the inclusion of a stabilizer component significantly reduces manual manipulation by the interventional cardiologist, thereby reducing the complexity of catheter control during the injection process and increasing the system’s stability. Finally, safety is another cornerstone of the EndoWings® system. It allows for the setting of individualized needle depth based on the patient's myocardial thickness, reducing the risk of common complications such as perforation and leakage. We also validated real-time monitoring of the precise relationship between the catheter tip and corresponding structures within the LV cavity using echocardiography in a large animal model.

However, the maximum outer diameter of 18 F precludes the use of EndoWings® through a transradial approach. The design of an 18-F delivery system was primarily driven by our injection protocol, which aims to ensure stable and precise delivery of hydrogel into the mid-LV wall in a consecutive circumferential pattern. This predefined injection pattern necessitates superior maneuverability of the injection system, enabling extensive directional control of the catheter within the limited cardiac chamber space. Such control requires the system to possess high-torque transmission capabilities. Consequently, the distal end of the injection catheter has been designed with an adjustable bending section and a transition section. The bending section incorporates a composite tube with pull rings and traction wires to facilitate catheter tip bending. The transition section is structured with multiple layers, including a high-rigidity elastic layer, a metal braiding layer, an enhanced traction wire cavity, and an inner plastic tube layer, to augment torque transmission and effectively resist deformation from traction. This complex design enables the torque ratio from the distal to the proximal end of the injection catheter to exceed 270°/360°. Furthermore, during our animal experiments, we observed that the catheter often assumes an eccentric position post-passage through the aortic valve, impeding valve closure and leading to regurgitation. To address this issue, our GC is also designed with an adjustable bending feature to avoid compromising aortic valve function. However, this enhanced functionality necessitates an increase in the GC’s outer diameter to accommodate the internal traction mechanism. However, the size of the GC is clinically acceptable based on global experience in structural heart interventions. This is due to the fact that in clinical practice, effective hemostasis at the puncture site can be achieved with a single ProGlide after the removal of an 18-F interventional device.

## Limitation

One of the limitations of this preclinical study is the use of healthy large animal models, which may not fully replicate the pathological conditions observed in patients with heart failure. Although the structure of the swine heart is similar to that of the human heart, the function of the swine heart is normal. This contrasts with patients with chronic heart failure, in whom the left ventricular cavity would be larger than in the animal models used in our study. In addition, because the primary objective of this study was not to validate the hypothesis that TEAi could treat heart failure, using a healthy swine model limits our ability to evaluate the therapeutic efficacy of this treatment for heart failure. Another limitation is that this study did not evaluate the long-term effects of treatment on cardiac function. Finally, this study is limited by the lack of continuous ECG recordings during postoperative follow-up due to the lack of appropriate monitoring equipment. As an alternative, we used routine daily 12-lead ECG monitoring. Although no arrhythmias were detected and there were no cases of sudden death in the pigs, it must be acknowledged that this approach may have missed potential transient arrhythmias.

## Conclusion

The preclinical model in large animals demonstrated that the use of the novel technological combination of shear-thinning alginate hydrogel XDROP® with the dedicated EndoWings® system for transendocardial alginate hydrogel injection (TEAi) is feasible and safe. These findings support further clinical studies of TEAi in treating heart failure.

## Supplementary Information

Below is the link to the electronic supplementary material.

Supplementary file1 (DOCX 8107 KB)

Supplementary file2 (MP4 39310 KB)

## Data Availability

The datasets generated and/or analyzed during the current study are available from the corresponding author (L.T.) upon reasonable request.

## Abbreviations

AH: Alginate hydrogel
EF: Ejection fraction
LV: Left ventricle
PM: Papillary muscles
TEE: Transesophageal echocardiography
TEAi: Transendocardial alginate injection
