# Supplementary material for: Percutaneous Alginate Hydrogel Endomyocardial Injection with a Novel Dedicated Catheter Delivery System: An Animal Feasibility Study
Source: J Cardiovasc Transl Res. 2024 Feb 20;17(4):842–50. doi: 10.1007/s12265-024-10497-8 (PMC11371841; doi:10.1007/s12265-024-10497-8)

**Supplementary Information**

**Novel Dedicated Catheter for Percutaneous Alginate Hydrogel Endomyocardial Injection: A Large Animal Feasibility Study**

Bo Wang^1,2^, Chao Gao^1^, D. Scott Lim^3^, Rutao Wang^1^, Cun-jun Zhu^1^, Yoshinobu Onuma^2^, Yunbing Wang^4^, Runlin Gao^5^, Patrick W.J.C. Serruys^2¶^, Randall J. Lee^6¶^, Ling Tao^1¶^

1. Department of Cardiology, Xijing Hospital, Fourth Military Medical University, Xi'an, China
2. Corrib Research Centre for Advanced Imaging and Core Laboratory, University of Galway, Galway, Ireland
3. Department of Medicine, Division of Cardiovascular Medicine, University of Virginia, Charlottesville, VA, USA
4. National Engineering Research Center for Biomaterials, Sichuan University, Sichuan, China
5. Fuwai Hospital, Chinese Academy of Medical Sciences & Peking Union Medical College, Beijing, China.
6. Department of Medicine, University of California-San Francisco, San Francisco, CA, USA

[Supplemental Figure 3](#_Toc156169691)

[Supplemental Figure 1 Scanning Electron Microscope (SEM) images of XDROP^®^. 3](#_Toc156169692)

[Supplemental Figure 2 Experiment system for assessment of XDROP^®^ aggregation and microscope images of the filter. 4](#_Toc156169693)

[Supplemental Figure 3 Hematoxylin and eosin staining of myocardium 3 days after injection demonstrating moderate mononuclear cell infiltration. A and B are images of HE staining of myocardium. C and D are images of Masson’s trichrome staining of myocardium. 5](#_Toc156169694)

[Supplemental Figure 4 Examination of porcine organs. 6](#_Toc156169695)

[Supplemental Figure 5 Hematoxylin and eosin (H&E) staining of other porcine organs. 7](#_Toc156169696)

# Supplemental Figure

## Supplemental Figure 1 Scanning Electron Microscope (SEM) images of XDROP^®^.

A, B and C are images of the XDROP^®^ structure before delivery via EndoWings^®^ as examined by SEM (800x, 400x, 200x); D, E and F are images of the XDROP^®^ structure after delivery via EndoWings^®^ as examined by SEM (800x, 400x, 200x).


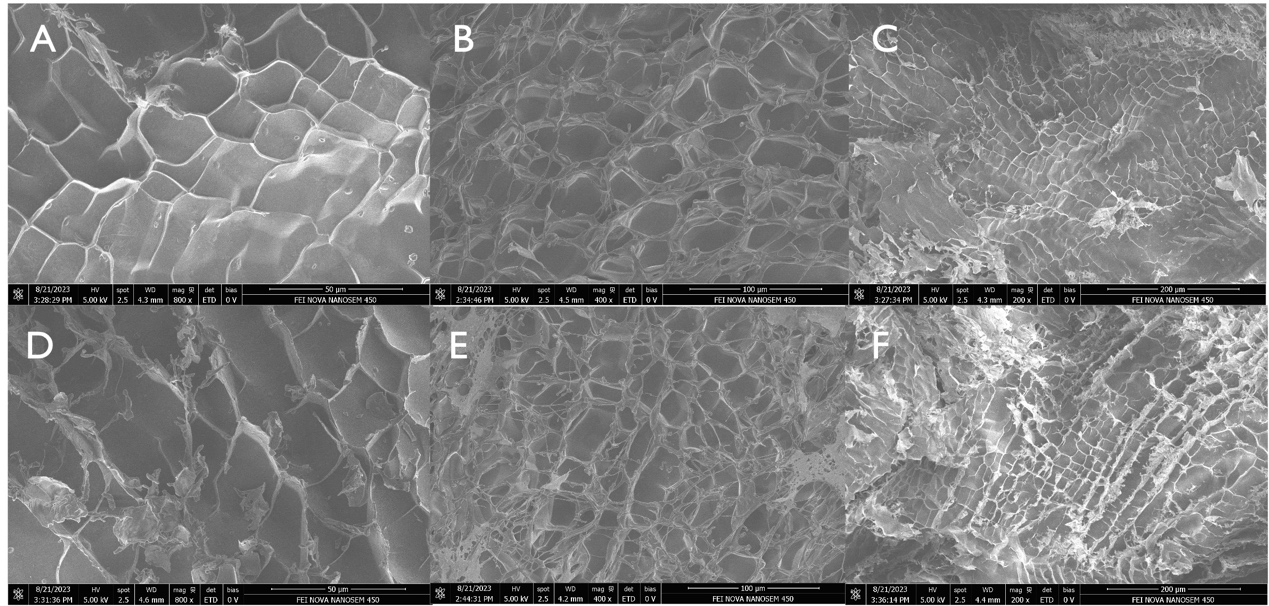


## Supplemental Figure 2 Experiment system for assessment of XDROP^®^ aggregation and microscope images of the filter.

**A** is the experiment system used to evaluate the XDROP^®^ aggregation in simulated human blood flow. B, C and D are images of the PEEK filter examined under a microscope (circulating for 1 hour, 12 hours, and 24 hours, separately).


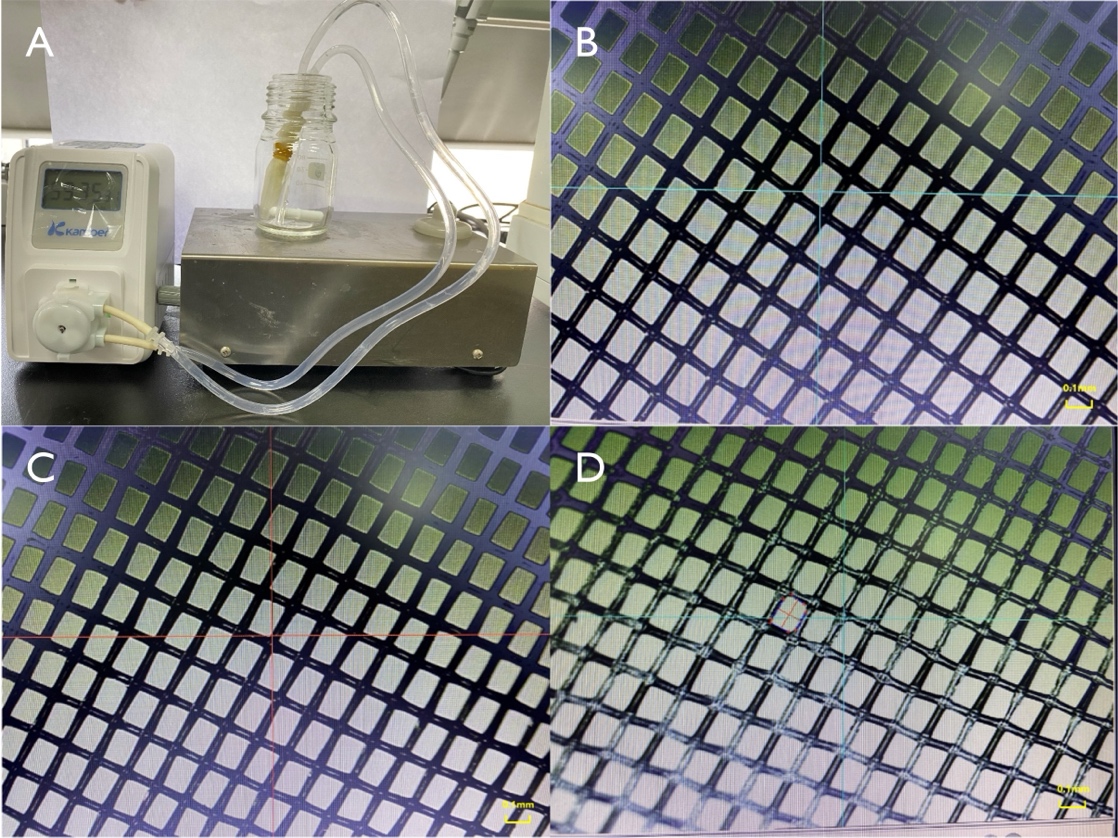


## Supplemental Figure 3 Hematoxylin and eosin staining of myocardium 3 days after injection demonstrating moderate mononuclear cell infiltration. A and B are images of HE staining of myocardium. C and D are images of Masson’s trichrome staining of myocardium.

## Supplemental Figure 4 Examination of porcine organs.


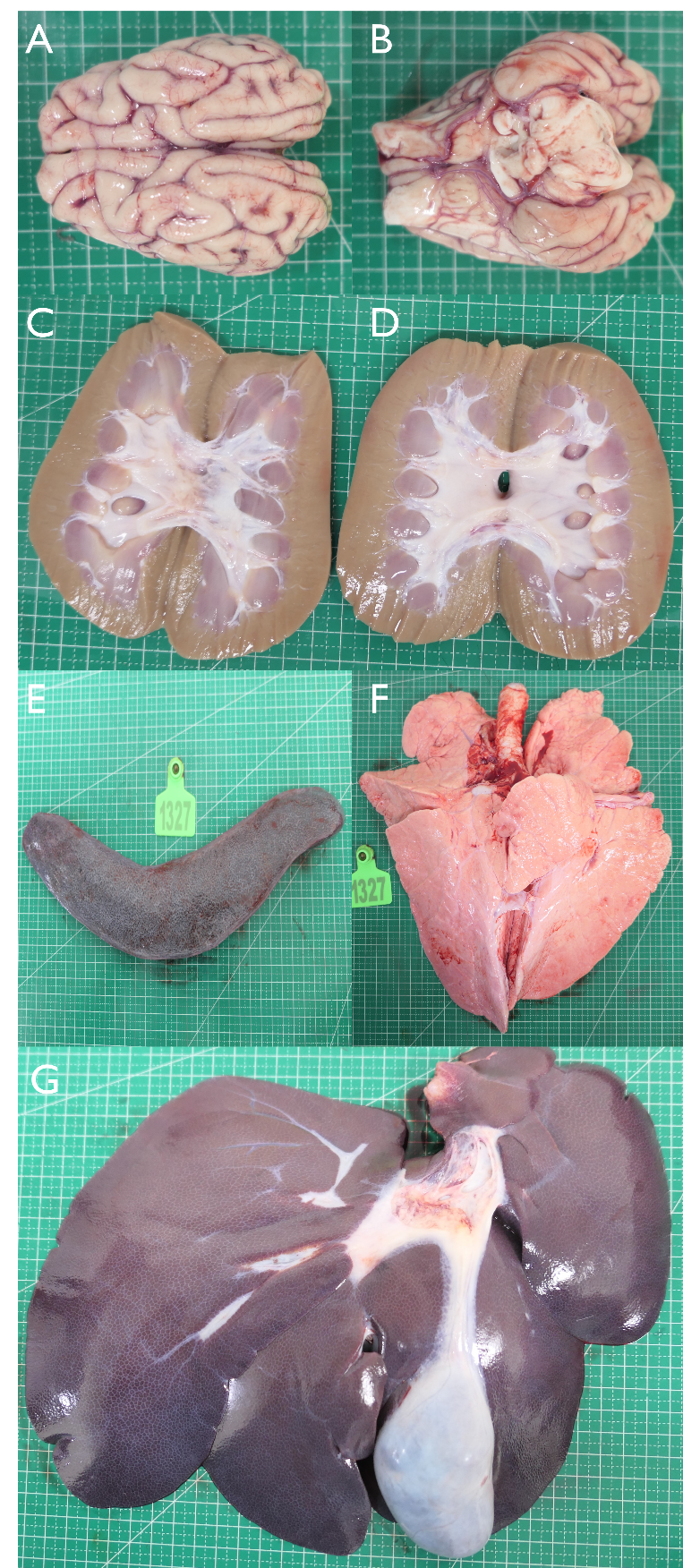
(A) Brain surface, (B) Cerebral circulation, (C) Left kidney, (D) Right kidney, (E) Spleen, (F) Lung, and (G) liver.

##

## Supplemental Figure 5 Hematoxylin and eosin (H&E) staining of other porcine organs.

(A) lung, (B) kidney, (C) liver, and (D) spleen.


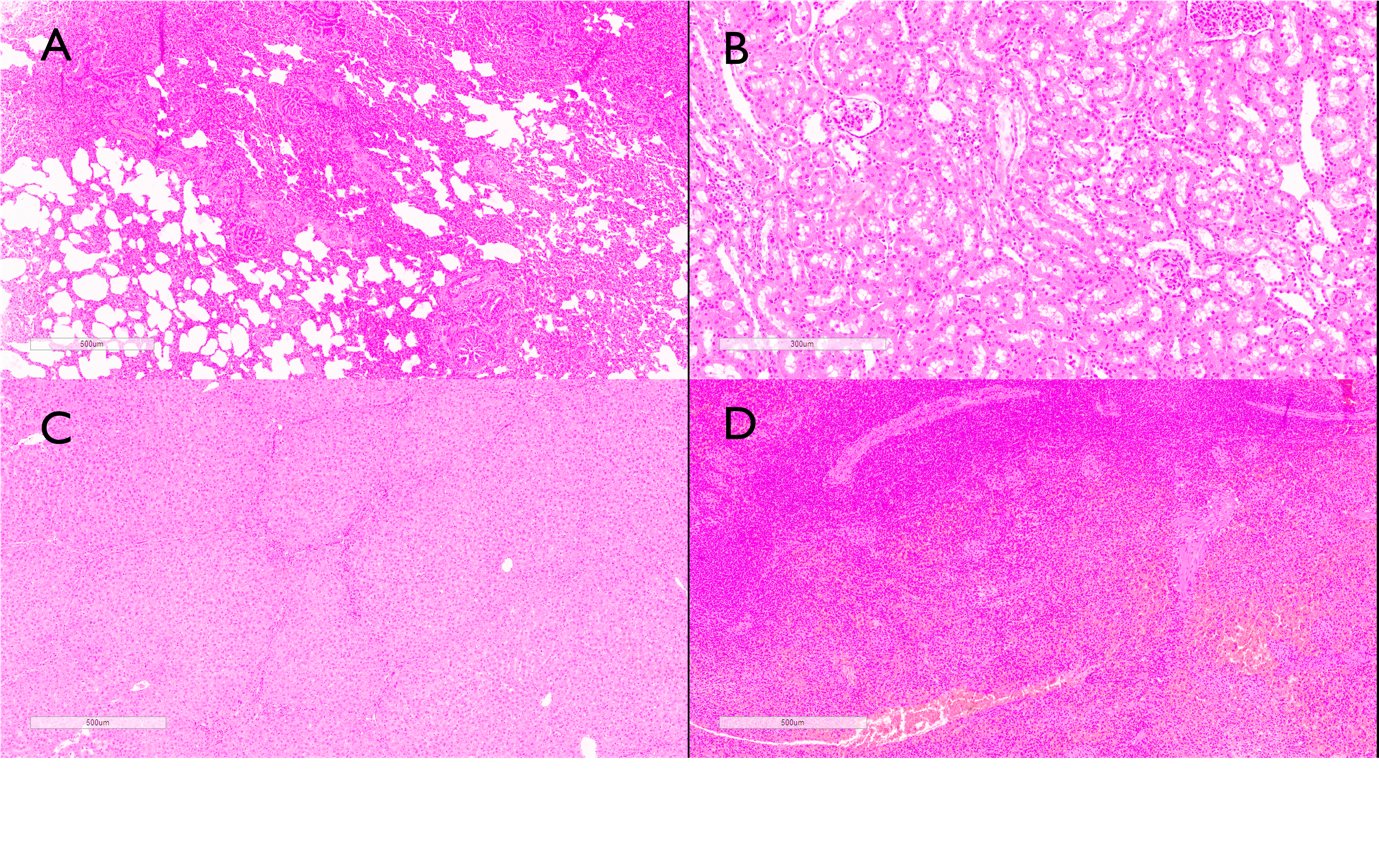

Supplement: Supplementary file 1 — Supplementary file1 (DOCX 8107 KB) [file 12265_2024_10497_MOESM1_ESM.docx]
